# A randomised, crossover, clinical study to assess nicotine pharmacokinetics and subjective effects of the BIDI^®^ stick ENDS compared with combustible cigarettes and a comparator ENDS in adult smokers

**DOI:** 10.1186/s12954-022-00638-0

**Published:** 2022-06-02

**Authors:** Ian M. Fearon, Karin Gilligan, Ryan G. N. Seltzer, Willie McKinney

**Affiliations:** 1 whatIF? Consulting Ltd, The Crispin, Burr Street, Harwell, OX11 0DT UK; 2 McKinney Regulatory Science Advisors, LLC, 4940 Old Main Street, Unit 603, Henrico, VA 23231 USA; 3 Safety in Numbers, LLC, 8110 S Houghton Rd Ste 158-552, Tucson, AZ 85747 USA

**Keywords:** Nicotine, Pharmacokinetics, Subjective effects, Electronic nicotine delivery system, Cigarette, Smoking

## Abstract

**Background:**

Nicotine pharmacokinetic assessments of electronic nicotine delivery systems (ENDS) are crucial to understand their ability to provide an alternative to cigarette smoking. Subjective effects data also strongly contribute to this understanding. The BIDI^®^ Stick is a disposable ENDS product which contains 59 mg/ml nicotine benzoate salt and various flavours.

**Methods:**

In this study, we assessed nicotine pharmacokinetics and subjective effects of 6 flavour variants of BIDI^®^ Stick ENDS in adult smokers, compared to cigarettes and a comparator ENDS product. During each of eight study visits, 18 volunteer smoker subjects randomly used one of either their usual brand (UB) of cigarette, a BIDI^®^ Stick ENDS, or a comparator ENDS (JUUL 59 mg/ml nicotine with Virginia Tobacco flavour), during both controlled (10 puffs, 30 s apart) and ad libitum (60 min) puffing sessions. Blood samples were collected at various time points and subjective effects questionnaires were administered.

**Results:**

Mean [SD] plasma nicotine C_max 0–120_ was not significantly different between BIDI^®^ Stick ENDS with any flavour (range 15.3 [9.90] ng/ml for BIDI^®^ Stick Winter to 17.6 [9.00] ng/ml for BIDI^®^ Stick Classic) and UB cigarettes (16.2 [9.17] ng/ml). Mean [SD] AUC_0-120_ (range 569.7 [327.29] to 628.6 [408.99]  min*ng/ml for BIDI^®^ Stick ENDS and 747.1 [325.48]  min*ng/ml for UB cigarettes) and median T_max 0–120_ (range 5-7 min for all BIDI^®^ Stick ENDS and UB cigarettes) values were also not significantly different between BIDI^®^ Stick ENDS and UB cigarettes, while subjective effects measures were also similar between BIDI^®^ Stick ENDS and UB cigarettes. Mean [SD] plasma nicotine C_max 0–120_, AUC_0-120_, and median T_max 0–120_ were 6.8 [4.13] ng/ml, 243.6 [179.04] min*ng/ml, and 5 min, respectively, for JUUL ENDS. These values were significantly different compared with those for all BIDI^®^ Stick ENDS and UB cigarettes for both C_max 0–120_ and AUC_0-120_ but not for T_max 0–120_.

**Conclusions:**

BIDI^®^ Stick ENDS delivered nicotine to users comparably to their UB combustible cigarette and higher than JUUL ENDS, and also elicited similar subjective effects such as satisfaction and relief. Thus, the BIDI^®^ Stick ENDS may be a satisfying alternative to cigarettes among current smokers and may support their transitioning away from cigarette smoking.

***Trial registration*:**

ClinicalTrials.gov (identifier number NCT05072925).

**Supplementary Information:**

The online version contains supplementary material available at 10.1186/s12954-022-00638-0.

## Background

Cigarette smoking is the leading preventable cause of morbidity and mortality worldwide and is the primary causative factor in the deaths of more than 7 million smokers annually [1]. A number of serious human diseases are caused by cigarette smoking, including heart disease, lung disease, and lung cancer, which arise due to a smokers’ inhalation of toxic chemicals formed during the combustion of tobacco [2–4]. Cigarette smoke contains approximately 6,500 identified chemicals [3] and a number of these chemicals have a demonstrated association with the development of specific smoking-related diseases [5]. For smokers, the best possible means of reducing the risk to their health is to quit smoking [6] and large numbers of adult smokers report such a desire to stop smoking. However, the addictive nature of cigarette smoking means that quitting smoking is inherently difficult. Unfortunately, less than 10% of adult smokers actually manage to stop smoking annually [7].

Forms of nicotine delivery that satisfy a smokers’ desire for nicotine and reduce or eliminate exposure to tar and harmful toxicants found in cigarette smoke have been suggested since the 1970s as a means to reduce smoking-related health risks [8]. Regarding smokers who are either unable or unwilling to quit smoking, a number of public health bodies such as Public Health England, the UK Royal College of Physicians, the New Zealand Ministry of Health and Health Canada, have proposed that reduced-exposure products may provide a less harmful alternative to combustible cigarettes and support efforts to reduce the global burden of cigarette smoking [9–11]. E-cigarettes are a form of electronic nicotine delivery systems (ENDS) which generate an aerosol via electrical heating of an e-liquid that most commonly contains nicotine [12, 13]. Since the heating temperature required to aerosolise e-liquids is much lower than the smoke-producing temperature developed during the combustion of tobacco leaves in conventional cigarettes, ENDS aerosols contain far fewer and substantially lower levels of harmful toxicants compared with cigarette smoke [14–16]. Therefore, exposure to cigarette smoke toxicants is either greatly reduced or absent in smokers who completely switch to ENDS [17–22]. In many instances, biomarkers of exposure are at levels seen with smoking abstinence [17, 19] or in non-smokers. This exposure reduction has the potential to reduce the risk of tobacco-related disease in smokers who completely switch to using ENDS. Consequently, some public health bodies, including Public Health England, have proposed the use of ENDS as a potentially reduced-harm alternative to cigarette smoking for adult smokers [9], and particularly those who have been unable to quit by other means. Furthermore, a growing body of the literature indicates that ENDS have the potential to support smoking cessation [23], particularly in those who use ENDS daily and non-intermittently [24, 25].

It has been suggested that nicotine delivery from ENDS is an important factor in determining their ability to facilitate smokers’ switching away from cigarette smoking [26–28]. Nicotine-containing smoking cessation products such as nicotine gum that have higher nicotine content (e.g. 4 mg gum compared with 2 mg gum) deliver greater amounts of nicotine to users, produce greater satisfying and reinforcing effects, and are more effective in promoting smoking cessation [26, 29–31] particularly among highly dependent smokers [26, 32, 33]. For ENDS,  greater nicotine delivery is associated with greater reductions in urges to smoke as well as other beneficial subjective effects such as greater satisfaction, liking and reductions in withdrawal symptoms, and greater reductions in exposure to cigarette smoketoxicants [34–36].

The BIDI^®^ Stick is a disposable ENDS that contains an e-liquid with 59 mg/ml nicotine in the form of a nicotine benzoate salt and a variety of flavours, which has been marketed in the USA as an alternative to cigarette smoking for adult smokers since 2014. While the nicotine pharmacokinetic profile of various types of ENDS products has been reported in the literature [13, 37, 38], including disposable ENDS [39], no studies have yet examined nicotine pharmacokinetics for disposable ENDS with a high concentration of nicotine salt in the e-liquid. In this paper, we describe findings from a clinical study assessing nicotine pharmacokinetics and subjective effects of the BIDI^®^ Stick ENDS with various flavours, compared to combustible cigarettes and a comparator pod-based (JUUL) ENDS.

## Methods

This study was an open-label, randomised, crossover, clinical study in which healthy adult smokers were assigned to use one of eight investigational products at each clinic visit and according to a pre-determined randomisation schedule. The study was conducted in July and August 2021 at the facilities of MTZ Clinical Research Sp. z.o.o., Warsaw, Poland, in accordance with the principles of International Conference on Harmonisation Harmonised Tripartite Guideline for Good Clinical Practice (GCP) and the Declaration of Helsinki. GCP compliance was assured by both a pre-study GCP audit by an independent auditor and by frequent monitoring visits during study conduct by an independent Clinical Research Associate. Ethics approval was received from the Ethics Committee of the District Medical Board in Warsaw (Resolution 15/21, 29 April 2021). All subjects received financial remuneration for their participation in the study, which was approved by the ethics committee. The study was registered on the ClinicalTrials.gov repository (identifier number NCT05072925).

### Subjects

Subjects were adults aged 21–65 years inclusive and were current smokers of at least 10 factory-manufactured cigarettes a day with a Federal Trade Commission tar yield of 8-10 mg, had been smoking cigarettes for at least 12 months, and may have been dual users of ENDS. At a screening visit, which took place no more than 28 days before the first study visit, potential subjects provided written consent on an ethics committee-approved informed consent form. At this visit, a review of the potential subjects’ medical history, a physical examination, clinical laboratory assessments, an electrocardiogram (ECG), vital signs measurements, a urine pregnancy test (female subjects only), and a chest X-ray were performed to ensure that potential subjects were healthy. Urinary cotinine (≥ 200 ng/mL) and exhaled carbon monoxide (eCO; > 10 ppm) were also assessed to confirm cigarette smoking status, and a urine screen for drugs of abuse was performed. Subjects’ cigarette smoking and nicotine product use history was captured, and the Fagerström Test for Cigarette Dependence (FTCD) [40] was administered.

Female subjects were ineligible if they were pregnant or breastfeeding and were required to practice a reliable method of contraception for the duration of the study. Exclusion criteria also included any clinically relevant medical or psychiatric disorder, abnormal findings in the physical examination, clinical laboratory assessments, ECG or chest X-ray, or a positive screen for drugs of abuse. Potential subjects who had a positive text for SARS-CoV-2 (COVID-19) or displayed any symptoms indicative of active SARS-CoV-2 infection were also excluded from the study.

### Study products

The BIDI^®^ Stick is a completely self-contained disposable, non-rechargeable ENDS device (see Additional file 1: Figure S1) containing 1.4 ml of e-liquid with a 6% w/v nicotine concentration (i.e. 59 mg/ml). Similar to other ENDS e-liquids, the BIDI^®^ Stick e-liquid also contains propylene glycol (25–35% w/v), vegetable glycerol (26–38% w/v), benzoic acid (7.2% w/v), and flavours. The device power source is a lithium-ion rechargeable battery cell with a capacity of 280mAh which is sufficient to last until the e-liquid in each BIDI^®^ Stick is completely consumed.

Six BIDI^®^ Stick ENDS, each containing 59 mg/ml nicotine benzoate salt and different flavours, were assessed in the study. The specific products assessed, and the flavour types for each variant, were BIDI^®^ Stick Arctic (mint and menthol), Classic (tobacco), Zest (melon, pineapple and banana), Regal (dragonfruit and strawberry), Winter (watermelon, melon, and menthol), and Solar (strawberry and blueberry). A comparator ENDS product, the JUUL pod system ENDS with 59 mg/ml nicotine benzoate salt and Virginia Tobacco flavour, was also assessed. All subjects provided their usual brand (UB) of combustible cigarette for use as a reference cigarette.

### Randomisation procedure

Randomisation sequences were prepared by MTZ Clinical Research Sp. z.o.o. and were produced using a block randomisation (Williams) procedure (18 subjects randomised to the 14 treatment sequences, size of block equal to 1) for 7 treatments in 7 periods (i.e. generation of a Latin-square design, where every treatment followed every other treatment the same number of times). Equal allocation of subjects to each sequence was ensured.

### Study procedures

At screening, subjects underwent numerous assessments outlined above to assure their health status. Subjects who passed all screening assessments and provided written informed consent visited the clinic site on eight separate occasions, with each clinic visit separated by at least two days. At the first of these visits, subjects underwent nicotine pharmacokinetic and subjective effects assessments with their usual brand (UB) of combustible cigarette. Prior to each subsequent visit, subjects were provided with a supply of either the BIDI^®^ Stick ENDS or the JUUL ENDS they were to use at their next clinic visit according to the randomisation schedule, to use at home for a familiarisation period of at least two days. At each clinic visit, subjects used their randomly assigned product and underwent nicotine pharmacokinetic and subjective effects assessments. Prior to each clinic visit, subjects were instructed to abstain from the use of any nicotine-containing products for a period of at least 12 h. Compliance with this instruction was assessed by measuring eCO with a cut-off level of < 15 ppm [26, 41, 42]. After the final clinic visit, subjects were discharged from the clinic after all nicotine pharmacokinetic and subjective effects assessments were completed. Subjects were contacted by telephone no longer than one week after the final study visit to capture any post-study adverse events (AEs).

### Nicotine pharmacokinetics

During the first clinic visit (Visit 2), subjects smoked their UB combustible cigarette during two use sessions. In the first session, subjects smoked a single combustible cigarette by taking 10 puffs with each puff 30 s apart (controlled puffing). Blood samples (4 mL) were obtained for plasma nicotine analysis at -5, 3, 5, 7, 10, 15, 30, 45, 60, 75, and 120 min relative to the first puff on the cigarette. In the second session, which began immediately after the last (120 min) blood draw, subjects were allowed to take ad libitum puffs on their UB cigarette for a period of 60 min. During this ad libitum session, subjects were allowed to smoke as many cigarettes as they liked. A blood sample for nicotine pharmacokinetic analysis was drawn at the end of the session (i.e. at 180 min). At subsequent visits, subjects used their assigned ENDS product following the same procedures.

Blood samples (4 ml) for plasma nicotine analysis were drawn into dipotassium ethylenediaminetetraacetic acid (K_2_ EDTA) vacutainer tubes via an intravenous catheter port. No later than 90 min after collection, samples were centrifuged at 1500 RPM at 4 °C for 10 min. The plasma fraction was transferred to two sterile polypropylene screw cap tubes and stored frozen at − 20 °C within 120 min of collection. Plasma samples were shipped on dry ice to a commercial bioanalytical laboratory (Altasciences Company Inc., Laval, Quebec, Canada). Nicotine levels were assessed with a validated reversed-phase HPLC with MS/MS method, using an AB Sciex API 5000 quadrupole mass spectrometer and a Turbo V ion source with ES probe and operating in positive ion mode. The lower limit of quantification (LLOQ) for this assay was 0.200 ng/ml, and the upper limit of quantification (ULOQ) was 100.000 ng/ml.

### Mass loss

The BIDI^®^ Stick and JUUL ENDS were weighed before and after both the controlled and ad libitum use sessions. Mass loss was then calculated by subtracting the mass post-use from the mass pre-use.

### Subjective effects assessments

At the end of the ad libitum puffing session, subjects completed the 21-item Product Evaluation Scale (PES) [43] for which responses were recorded on a 7-point Likert scale ranging from “Not at all” to “Extremely”.

### Statistical analyses

Since this study was the first to examine nicotine pharmacokinetics in subjects using BIDI^®^ Stick ENDS, no formal power calculations were performed. The sample size is typical of other studies reported in the literature examining the pharmacokinetics and subjective effects of different tobacco/nicotine products [37], and a sample size of 18 subjects was determined adequate to meet the study objectives.

Descriptive statistics for pharmacokinetic parameters, including baseline-adjusted maximum plasma nicotine concentration between 0 and 120 min (C_max 0–120_); time to maximum plasma nicotine concentration following controlled puffing (T_max 0–120_); and baseline-adjusted area under the plasma nicotine concentration–time curve at 120 min (AUC_0-120_), were summarised for each study product. AUC_0-120_ was calculated using a linear trapezoidal method. Following baseline adjustment, if any component of the AUC_0-120_ fell below zero, that component was excluded from the overall AUC value.

For inferential statistical analyses, linear mixed models were used to test differences in log-transformed C_max_ _0–120_ and AUC_0-120_ values between BIDI^®^ Stick ENDS, JUUL, and UB cigarettes. Subject was included as a random effect. The sequence of product used was initially specified as a random effect, but the models produced non-positive definite G matrices and so this variable was removed as a random effect. Model parameter estimates were exponentiated back to their original scale and used to create 90% confidence intervals for the ratio of geometric, least-squares means. Statistically significant differences between test products were determined if the 90% confidence interval range did not include the value 1.00. Proportional odds generalised linear mixed models were used to test differences in T_max_ values between BIDI^®^ Stick ENDS, JUUL, and UB cigarettes. The subject and sequence order of the product used were specified as random effects. Statistical significance was determined for 90% odds ratio confidence intervals that did not contain the value 1.00.

The PES was analysed by assessing four composite subscales: (1) “satisfaction”; (2) “psychological reward”; (3) “aversion”; and (4) “relief” [43]. PES subscale scores were summarised using descriptive statistics for each study product, and post hoc pairwise comparisons between study products were made using linear mixed effects models with the subject specified as a random effect.

Statistical analyses were performed using SAS Version 9.4 (Cary, NC, USA) with alpha = 0.05 (2-tailed).

### Safety assessments

Safety and tolerability were assessed by collecting information concerning the incidence, nature, and severity of any AEs experienced by subjects. Vital signs (blood pressure and heart rate) were also routinely monitored during study visits.

## Results

### Study population

Of 41 subjects who were screened, 18 (43.9%) met all of the inclusion criteria and none of the exclusion criteria and were enrolled into the study. Seventeen subjects attended the clinical site for all eight study visits and used their randomly assigned study product in the product use sessions; one subject was discontinued after Visit 2 (see later section on safety assessments). Brief demographic details of the 18 subjects are provided in Additional file 1: Table S1. Subjects’ mean [SD] age was 39.2 [9.21] years, approximately two-thirds were male, and all were of Caucasian race. On average, subjects FTCD score was 5.8 [1.66] and subjects usually smoked on average 16.6 [4.82] cigarettes per day and had been smoking for an average of 19.3 [7.66] years. Two subjects had prior experience of ENDS use, but neither these nor any other subjects were currently using ENDS.

### Nicotine pharmacokinetics

**Fig. 1:**
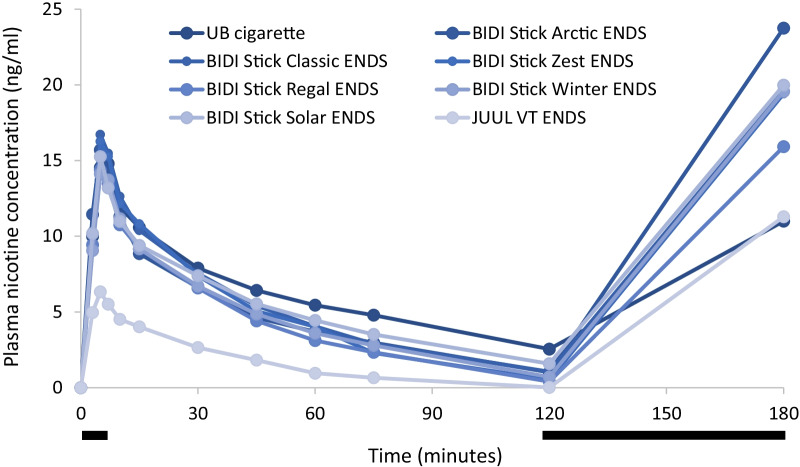
Meanbaseline-adjusted plasma nicotine concentration by time. *N* = 17–18 in each case. Solid black bars below the plot indicate the controlled (0–5 min) and ad libitum (120–180 min) puffing sessions. Errors bars have been omitted for clarity; for variability estimates, refer to Table 1. UB, usual brand; VT, Virginia Tobacco

During use of all study products in the controlled puffing session,plasma nicotine levels rose rapidly (Fig. 1). The mean [SD] maximum plasma nicotine concentration reached following this session (C_max 0–120_) was 16.2 [9.17] ng/ml for subjects’ UB combustible cigarette (Table 1). C_max 0–120_ values were not significantly different (Tables 1 and 2) for each of the BIDI^®^ Stick ENDS assessed compared both with each other and with UB cigarettes and ranged from 15.3 [9.90] ng/ml for BIDI^®^ Stick Winter to 17.6 [9.00] ng/ml for BIDI^®^ Stick Classic. C_max 0–120_ for the comparator ENDS product (JUUL Virginia Tobacco) was 6.8 [4.13] ng/ml, which was significantly lower than that for either UB cigarettes or any of the BIDI^®^ Stick ENDS (Table 2). Similar to C_max 0–120_, area under the plasma nicotine concentration–time curve between 0 and 120 min (AUC_0-120_) values for any of the BIDI^®^ Stick ENDS were not significantly different than that for UB cigarettes, while AUC_0-120_ for the comparator (JUUL) ENDS product was significantly lower than that for both UB cigarettes and any of the BIDI^®^ Stick ENDS (Tables 1 and 2). Mean time to maximum plasma nicotine concentration values for the controlled puffing session (T_max 0–120_) ranged from 6.0 min for both the BIDI^®^ Stick Regal and Winter ENDS (SDs 1.58 and 1.41, respectively) to 6.8 [2.51] min for BIDI^®^ Stick Zest (Table 1). Mean T_max 0–120_ for the JUUL ENDS was 5.9 [1.73] min. There were no statistically significant differences in T_max 0–120_ between any of the study products.

**Table 1:** Nicotine pharmacokinetic parameters for BIDI stick^®^ ENDS and comparator products

	UB cigarette	BIDI^®^ Stick Arctic	BIDI^®^ Stick Classic	BIDI^®^ Stick Regal	BIDI^®^ Stick Solar	BIDI^®^ Stick Winter	BIDI^®^ Stick Zest	JUUL VT
C_max 0–120_ (ng/mL)								
Mean [SD]	16.2 [9.17]	16.8 [9.71]	17.6 [9.00]	15.6 [8.72]	16.0 [11.73]	15.3 [9.90]	17.2 [10.30]	6.8 [4.13]
Geometric mean [SD]	14.0 [1.74]	13.5 [2.18]	15.5 [1.68]	13.4 [1.80]	12.6 [2.07]	13.1 [1.76]	14.7 [1.77]	5.7 [1.89]
Median	13.9	16.6	13.3	13.7	11.1	12.0	11.6	5.7
Min, max	4.7, 40.6	1.4, 36.8	7.1, 33.9	3.8, 37.0	2.6, 41.2	4.4–44.6	5.4–42.7	1.8–14.8
AUC_0-120_ (min*ng/mL)								
Mean [SD]	747.1 [325.48]	592.0 [254.76]	616.9 [296.45]	572.4 [315.83]	628.6 [408.99]	569.7 [327.29]	620.3 [283.86]	243.6 [179.04]
Geometric mean [SD]	679.1 [1.59]	464.2 [2.94]	560.2 [1.57]	489.5 [1.84]	511.7 [2.07]	480.4 [1.89]	558.8 [1.65]	161.4 [3.06]
Median	665.5	602.4	607.2	582.3	532.9	462.6	558.3	245.4
Min, max	233.4, 1448.4	8.64, 1118.8	248.1, 1491.0	137.1, 1412.1	61.6, 1679.2	111.7, 1206.6	136.8, 1312.7	8.9, 588.2
T_max 0–120_ (min)								
Mean [SD]	6.7 [2.74]	6.2 [1.39]	6.1 [1.41]	6.0 [1.58]	6.1 [1.58]	6.0 [1.41]	6.8 [2.51]	5.9 [1.73]
Median	6	7	5	5	7	5	7	5
Min, max	3, 15	5, 10	5, 10	3, 10	3, 10	5, 10	5, 15	3, 10
C_max 120–180_ (ng/ml)								
Mean [SD]	11.0 [5.30]	23.7 [12.67]	19.6 [10.43]	15.9 [13.29]	20.0 [11.73]	19.6 [14.67]	19.4 [10.21]	11.3 [10.85]
Geometric mean [SD]	10.04 [1.53]	18.61 [2.09]	17.32 [1.67]	12.44 [2.54]	16.96 [1.82]	16.86 [2.03]	17.56 [1.78]	8.52 [2.37]
Median	9.3	28.8	18.4	11.4	20.1	15.0	16.3	6.8
Min, max	4.9, 25.6	3.5, 38.4	8.5, 42.7	0.9, 48.9	7.4, 44.2	4.9, 50.1	5.3, 40.6	1.7, 41.2

*N* = 17–18 in each case

*UB* usual brand; *SD* standard deviation; *VT* Virginia Tobacco; *min* minimum; and *max* maximum

**Table 2:** Statistical comparison of nicotine pharmacokinetic parameters

	BIDI^®^ Stick Arctic	BIDI^®^ Stick Classic	BIDI^®^ Stick Regal	BIDI^®^ Stick Solar	BIDI^®^ Stick Winter	BIDI^®^ Stick Zest	JUUL VT
C_max 0–120_ LS Means [90% CI]^a^							
BIDI Stick^®^ Classic ENDS	0.89 [0.71,1.12]	–	–	–	–	–	–
BIDI Stick^®^ Regal ENDS	1.01 [0.80,1.27]	1.13 [0.90,1.43]	–	–	–	–	–
BIDI Stick^®^ Solar ENDS	1.07 [0.85,1.35]	1.20 [0.95,1.51]	1.06 [0.84,1.33]	–	–	–	–
BIDI Stick^®^ Winter ENDS	1.05 [0.83,1.33]	1.18 [0.93,1.49]	1.04 [0.82,1.31]	0.98 [0.78,1.24]	–	–	–
BIDI Stick^®^ Zest ENDS	0.94 [0.75,1.19]	1.06 [0.84,1.33]	0.93 [0.74,1.18]	0.88 [0.70,1.11]	0.89 [0.71,1.13]	–	–
JUUL VT ENDS	**2.44 [1.93,3.08]**	**2.74 [2.17,3.45]**	**2.42 [1.91,3.05]**	**2.28 [1.81,2.88]**	**2.32 [1.84,2.93]**	**2.59 [2.06,3.27]**	–
UB cigarette	1.00 [0.72,1.38]	1.12 [0.81,1.55]	0.99 [0.71,1.37]	0.93 [0.67,1.29]	0.95 [0.68,1.32]	1.06 [0.77,1.47]	**0.41 [0.30,0.57]**
AUC_0-120_ LS Means [90% CI]^a^							
BIDI Stick^®^ Classic ENDS	0.83 [0.63,1.09]	–	–	–	–	–	–
BIDI Stick^®^ Regal ENDS	0.97 [0.73,1.28]	1.17 [0.88,1.55]	–	–	–	–	–
BIDI Stick^®^ Solar ENDS	0.94 [0.71,1.24]	1.13 [0.85,1.5]	0.97 [0.73,1.28]	–	–	–	–
BIDI Stick^®^ Winter ENDS	0.99 [0.75,1.31]	1.20 [0.91,1.59]	1.02 [0.77,1.36]	1.06 [0.8,1.41]	–	–	–
BIDI Stick^®^ Zest ENDS	0.84 [0.63,1.11]	1.01 [0.76,1.34]	0.86 [0.65,1.14]	0.89 [0.67,1.18]	0.84 [0.64,1.11]	–	–
JUUL VT ENDS	**2.93 [2.21,3.88]**	**3.54 [2.68,4.69]**	**3.03 [2.29,4.01]**	**3.13 [2.37,4.15]**	**2.95 [2.23,3.91]**	**3.51 [2.65,4.64]**	–
UB cigarette	0.70 [0.47,1.04]	0.84 [0.57,1.25]	0.72 [0.49,1.07]	0.75 [0.5,1.11]	0.70 [0.48,1.04]	0.84 [0.56,1.24]	**0.24 [0.16,0.35]**
T_max 0–120_ Odds Ratios [90% CI]^b^							
BIDI Stick^®^ Classic ENDS	0.79 [0.25,2.42]	–	–	–	–	–	–
BIDI Stick^®^ Regal ENDS	0.70 [0.23,2.16]	0.89 [0.29,2.76]	–	–	–	–	–
BIDI Stick^®^ Solar ENDS	0.84 [0.27,2.58]	1.07 [0.35,3.30]	1.20 [0.39,3.71]	–	–	–	–
BIDI Stick^®^ Winter ENDS	0.65 [0.21,2.02]	0.83 [0.27,2.58]	0.93 [0.30,2.90]	0.78 [0.25,2.41]	–	–	–
BIDI Stick^®^ Zest ENDS	1.40 [0.46,4.29]	1.79 [0.58,5.49]	2.01 [0.65,6.19]	1.67 [0.54,5.13]	2.15 [0.70,6.65]	–	–
JUUL VT ENDS	0.59 [0.19,1.82]	0.75 [0.24,2.33]	0.84 [0.27,2.62]	0.70 [0.23,2.18]	0.90 [0.29,2.81]	0.42 [0.13,1.30]	–
UB cigarette	1.08 [0.36,3.27]	1.38 [0.45,4.19]	1.54 [0.51,4.71]	1.29 [0.42,3.91]	1.66 [0.54,5.06]	0.77 [0.26,2.32]	1.84 [0.60,5.65]

Statistical significance is concluded if the CIs do not contain 1.00

*UB* usual brand; *LS* least square; *CI* confidence interval; and *VT* Virginia Tobacco. Values which were significantly different are in bold type

*N* = 17–18 in each case

^a^Back-transformed (exponentiated) linear mixed model parameter estimates used to create 90% CI ratios of geometric least-squares means between study products

^b^Odds ratios 90% CIs

During the ad libitum use session, plasma nicotine levels rose again for all study products (Fig. 1). While no formal statistical analysis was performed, C_max 120–180_ was highest for BIDI^®^ Stick Arctic ENDS and lowest for UB cigarettes and JUUL ENDS (Fig. 1 and Table 1).

### Mass loss

Mean [SD] mass loss from the BIDI^®^ Stick ENDS during the controlled use sessions (Additional file 1: Table S2) ranged from 0.029 [0.0158] g (BIDI^®^ Stick Arctic) to 0.084 [0.2124] g (BIDI Stick Regal). Mass loss from the JUUL ENDS during the controlled use session was 0.010 [0.0063] g. Mass loss from the BIDI^®^ Stick ENDS during the ad libitum use sessions (Additional file 1: Table S2) ranged from 0.120 [0.0560] g (BIDI^®^ Stick Regal) to 0.146 [0.0776] g (BIDI Stick Arctic). Mass loss from the JUUL ENDS during the ad libitum use session was 0.083 [0.0728] g.

### Subjective effects

Analysis of the composite scores for the PES “relief”, “satisfaction”, and “aversion” subscales showed no statistically significant differences between any of the study products (Table 3). For the “psychological reward” subscale, no significant differences between UB cigarettes and BIDI^®^ Stick ENDS were observed for 5 of the flavour variants assessed, while for BIDI^®^ Stick Winter ENDS “psychological reward” was significantly lower (mean [SD] 3.53 [1.43]) than for UB cigarettes (4.45 [1.12]). The individual item “was it enough nicotine” item, which is a component of the “relief” subscale, was assessed individually. Mean score for this item was highest for the UB cigarette and lowest for JUUL Virginia Tobacco ENDS (Table 3). UB cigarette was significantly higher than BIDI^®^ Stick Arctic and JUUL Virginia Tobacco, while the JUUL ENDS was significantly lower than BIDI^®^ Stick Regal, Solar, and Zest.

**Table 3:** Product evaluation scale scores

	UB cigarette	BIDI.^®^ Stick Arctic	BIDI.^®^ Stick Classic	BIDI.^®^ Stick Regal	BIDI.^®^ Stick Solar	BIDI.^®^ Stick Winter	BIDI.^®^ Stick Zest	JUUL VT
*Relief*								
Mean [SD]	4.67 [1.13].^a^	4.44 [1.19].^a^	4.36 [1.13].^a^	4.66 [1.25].^a^	4.65 [1.17].^a^	4.15 [1.10].^a^	4.61 [1.29].^a^	4.24 [1.18].^a^
Median	4.70	4.60	4.60	4.80	4.80	4.00	4.60	4.20
Min to max	2.80–7.00	2.20–7.00	2.20–6.00	2.00–7.00	2.20–6.20	2.20–6.00	2.20–7.00	2.60–7.00
*Satisfaction*								
Mean [SD]	4.56 [1.47].^a^	4.76 [1.45].^a^	4.24 [1.44].^a^	4.74 [1.21].^a^	5.00 [1.37].^a^	4.50 [1.77].^a^	4.97 [1.22].^a^	4.22 [1.37].^a^
Median	4.13	5.50	4.50	4.50	5.00	5.25	5.00	4.00
Min to max	2.25–7.00	2.50–7.00	1.00–6.00	2.25–7.00	2.25–7.00	1.00–6.75	2.00–7.00	2.00–6.75
*Psychological Reward*								
Mean [SD]	4.54 [1.12].^b^	4.06 [1.34].^ab^	3.98 [1.48].^ab^	4.06 [1.31].^ab^	4.06 [1.03].^ab^	**3.53 [1.43]**.^**a**^	4.05 [1.47].^ab^	4.04 [1.27].^ab^
Median	4.60	4.00	4.00	4.20	4.20	4.00	4.00	3.60
Min to max	2.80–7.00	1.80–6.00	1.00–7.00	2.00–7.00	2.20–5.40	1.00–5.40	1.00–7.00	2.00–6.80
*Aversion*								
Mean [SD]	2.24 [1.49].^a^	2.10 [0.94].^a^	2.13 [1.10].^a^	2.49 [1.17].^a^	1.96 [1.12].^a^	2.03 [1.15].^a^	2.49 [1.52].^a^	2.12 [1.03].^a^
Median	1.88	2.00	1.75	2.50	1.50	1.50	2.25	2.00
Min to max	1.00–7.00	1.00–4.00	1.00–4.50	1.00–5.00	1.00–4.00	1.00–4.00	1.00–5.00	1.00–4.00
*Was it Enough Nicotine Item*								
Mean [SD]	5.39 [1.29].^d^	**4.47 [1.62]**.^**abc**^	4.76 [1.52].^abcd^	5.12 [1.54].^ad^	5.00 [1.27].^abd^	4.65 [1.54].^abcd^	4.88 [1.58].^abd^	**4.06 [1.85]**.^**c**^
Median	5.00	4.00	5.00	5.00	5.00	5.00	5.00	4.00
Min to max	3.00–7.00	1.00–7.00	1.00–7.00	2.00–7.00	3.00–7.00	1.00–7.00	1.00–7.00	1.00–7.00

Pairwise comparisons were tested from the omnibus linear mixed model. Test products in the same row that do not share superscripts significantly differ (*p* < 0.05) based on a linear mixed model. Additionally, mean values in bold indicate significantly a significant difference compared to UB cigarettes. The enough nicotine individual item is a component of the “relief” subscale. All items were answered on seven-point response scales from 1 (“not at all”) to 7 (“extremely”)

*UB* usual brand; *Min* minimum; *max* maximum; *VT* Virginia Tobacco; and *SD* standard deviation

*N* = 17–18 in each case

### Safety assessments

No serious adverse events occurred during the study. A small number of adverse events occurred in some subjects, including headaches, dizziness, and events related to blood draws (e.g. bruising). These were all classed as either mild or moderate and quickly resolved without treatment. One subject withdrew from the study following their experiencing a number of adverse events (headache, nausea, decreased appetite, coughing) after the nicotine pharmacokinetic session at Visit 2 (UB cigarette smoking).

## Discussion

The primary finding from this clinical study was that the BIDI^®^ Stick ENDS delivered nicotine to users in a manner comparable to that from subject’s UB combustible cigarette. In terms of C_max_, AUC and T_max_, these parameters were not significantly different for any flavour of BIDI^®^ Stick ENDS compared to the combustible cigarette. Such a finding is unique in the literature for a disposable e-cigarette, although refillable tank-type ENDS devices and pod-based e-cigarettes have been found to deliver nicotine in a manner similar to [12, 13, 27, 28] or exceeding [44] that from combustible cigarettes. It has been proposed that providing sufficient nicotine delivery with either a greatly reduced, or absence of, exposure to harmful toxicants would be tolerated by smokers and thus may better serve tobacco harm reduction efforts by shifting smokers down the continuum of risk towards a less harmful tobacco product [8, 10] or helping them to stop smoking [27]. Furthermore, it has been acknowledged by the U.S. Food and Drug Administration that cigarette-like nicotine delivery from the heated tobacco product IQOS is potentially beneficial to smokers trying to switch since they are more likely to completely switch away from and not resume combustible cigarette smoking [10, 45], while a recent study concluded that an ENDS was most likely to help smokers reduce toxicant exposure and cigarette consumption when it was capable of delivering nicotine at levels similar to that of a cigarette [35]. In addition, it was proposed following a comparison of the nicotine delivery between US and European versions of the JUUL ENDS which differed in their e-liquid nicotine concentrations (59 mg/ml in the US version and 18 mg/ml in the European version) that nicotine delivery from the European version was not as effective and this may limit its potential in helping smokers stop smoking [28]. Effective nicotine delivery from non-inhaled smoking cessation products is also suggested to provide better assistance in smoking cessation [26, 30–33]. Overall therefore, our findings support that disposable e-cigarettes can, if consideration is given to product characteristics such as e-liquid nicotine concentration, inclusion of a protonating acid and providing sufficient power to the coil to generate aerosol, generate efficient nicotine delivery comparable to that from combustible cigarettes. These products may then be able to displace cigarette smoking and facilitate smokers switching to a form of nicotine intake with reduced exposure to harmful toxicants.

It has been suggested that high nicotine delivery from ENDS may be harmful if it leads to greater dependence [44], although a recent study [46] reported no differences in dependence between users of low, medium, and high strength nicotine e-liquids in either pod-based or disposable ENDS which presumably give rise to different nicotine exposures. Furthermore, from our nicotine pharmacokinetic and subjective effects findings it is unlikely that dependence on BIDI^®^ Stick ENDS would be greater than that of combustible cigarettes, and this is supported by the literature which suggests lower dependence on ENDS compared to cigarettes [47], although that analysis did not take e-liquid nicotine concentration into account and likely arose from an analysis of users of a diverse range of nicotine concentrations. A recent study also examined dependence among smokers who switched to using the JUUL ENDS, demonstrating no difference in dependence in users of either the 35 mg/ml or the 59 mg/ml nicotine concentrations, as well as demonstrating that regardless of nicotine concentration used, dependence on JUUL use was lower than dependence on cigarette smoking [48]. While it is unlikely therefore that dependence on using BIDI^®^ Stick ENDS would be higher than dependence on cigarette smoking, and may in fact be lower, this requires further assessment.

An interesting facet of our analyses of pharmacokinetic data is the finding of no difference in nicotine pharmacokinetics between different ENDS flavours. A small number of studies have assessed the impact of ENDS flavours on nicotine pharmacokinetics; one study reported an impact of flavours on C_max_ although the data appeared skewed by an abnormally high C_max_ for a cherry flavour which was likely due to the cherry e-liquid having a lower pH than the other liquids and much smaller differences were seen between other flavours with a similar pH [49], a small impact of certain flavours [49–51], or no impact [52, 53]. Our study data show that a comprehensive range of flavours, including mint/menthol and fruit flavours, did not differentially impact nicotine pharmacokinetics or abuse liability/dependence measures when compared to tobacco flavours.

In addition to comparable nicotine delivery to cigarettes, we observed comparable subjective effects following the use of BIDI^®^ Sticks ENDS. Plasma nicotine C_max_ in ENDS users correlates with satisfaction [34], while other subjective effects related to ENDS use are also indicators of the potential for ENDS to act as a viable alternative to cigarette smoking [54, 55]. This is likely of great importance when considering the harm reduction potential of ENDS. In this regard, a multidimensional framework for nicotine-containing products has been developed [54] which takes into account toxicity/harmfulness, appeal, and dependence. Using this framework, it has been suggested that the “sweet spot” for a nicotine product occurs when appeal and dependence are maximised and toxicity and harmfulness are minimised. Several studies have demonstrated reduced toxicant levels in ENDS emissions compared to cigarette smoke [9, 14–16]. In line with these findings, emissions testing of BIDI^®^ Stick ENDS demonstrated toxicant levels which were significantly less than those in cigarette smoke (unpublished data; see Additional file 1: Table S3). This supports a profile of lower toxicity and potential for inducing harm compared with cigarette smoking. Given this potential lower toxicity, along with evidence of comparable abuse liability/dependence potential (based on nicotine delivery and subjective effects) and comparable appeal (based on subjective effects findings), this suggests that the BIDI^®^ Stick ENDS have an appropriate balance of toxicity, appeal, and dependence and are a viable alternative to cigarette smoking which will likely have a positive impact on net population health [54].

Interpretation of the findings from this study may be subject to some limitations. Firstly, the study was conducted in a cohort of smokers in Poland, whereas BIDI^®^ Stick ENDS are currently only marketed in the USA. Thus, our nicotine pharmacokinetic and subjective effects data may not reflect those of a US smoker switching to using the study ENDS. However, this limitation is mitigated by the study inclusion criteria, which ensured that only smokers of high-yield cigarettes were eligible for entry into the study, an approach taken to more closely match the higher yield cigarettes more commonly smoked by US smokers [56, 57]. Furthermore, in the study a comparator ENDS (JUUL Virginia Tobacco) was included and our nicotine pharmacokinetic findings for both combustible cigarettes and JUUL Virginia Tobacco closely match those previously reported in similar studies in US smokers [50, 58]. Secondly, while our studies included a period in which study subjects were allowed to use the study products prior to their nicotine pharmacokinetic and subjective effects assessments, this period was short. It has been noted that nicotine delivery from ENDS may change over time as users become acclimatised to the devices [36, 59, 60], and therefore, our findings may not reflect nicotine delivery in an acclimatised BIDI^®^ Stick ENDS user. Thirdly, the study was of an open-label design, due primarily to the difficulty in blinding the subjects to the different study products (BIDI^®^ Stick ENDS, JUUL ENDS, and UB cigarettes) which were all very different in format. It was also not possible to blind the subjects to the ENDS flavours. Finally, the study sample size was potentially not sufficient to identify statistically significant differences in the subjective effects measures.

## Conclusions

In summary, nicotine pharmacokinetic assessments showed that the BIDI^®^ Stick ENDS delivered nicotine to users in a manner comparable to their UB combustible cigarette, while also inducing comparable subjective effects including satisfaction and relief. These findings support the BIDI^®^ Stick ENDS as a satisfying alternative for current smokers and may support their transitioning away from harmful cigarette smoking.

## Supplementary Information

**Additional file 1: Fig. S1.** Image of the BIDI^®^ Stick ENDS.** Table S1.** Demographic details for study subjects. ^a^Fagerström Test for Cigarette Dependence (FTCD) score at screening. ^b^Self-reported daily cigarette consumption at screening. Abbreviations: BMI, body mass index; min, minimum; max, maximum. **Table S2.** Mass loss from BIDI^®^ Stick and JUUL ENDS during controlled and* ad libitum* use sessions. Data are presented in grams (g). Abbreviations: Min, minimum; max, maximum; VT, Virginia Tobacco.** Table S3.** Levels of Harmful and Potentially Harmful Constituents (HPHCs) in BIDI^®^ Stick ENDS aerosol and 3R4F reference cigarette smoke. Data are per puff levels collected under International Organisation for Standardisation (ISO) conditions (55 ml puff volume, 2 second puff duration, 30 second interpuff interval) by a contract laboratory accredited under ISO 17025. Abbreviations: NA, not analysed; BDL, below detectable levels; NNK, Methyl[4-oxo-4-(pyridin-3-yl)butyl]nitrous amide; NNN, 3-[(2S)-1-Nitrosopyrrolidin-2-yl]pyridine.

## Data Availability

The datasets analysed during the study are available from the corresponding author on reasonable request.

## Abbreviations

AE: Adverse event
AUC: Area under the plasma nicotine concentration–time curve
CI: Confidence interval
C_max_: Maximum plasma nicotine concentration
ECG: Electrocardiogram
eCO: Exhaled carbon monoxide
ENDS: Electronic nicotine delivery systems
FTCD: Fagerstrom Test for Cigarette Dependence
GCP: Good clinical practice
HPLC: High-performance liquid chromatography
K_2_EDTA: Dipotassium ethylenediaminetetraacetic acid
LLOQ: Lower limit of quantification
PES: Product evaluation scale
RPM: Revolutions per minute
SD: Standard deviation
T_max_: Time of maximum plasma nicotine concentration
UB: Usual brand (of combustible cigarette)
UK: United Kingdom
ULOQ: Upper limit of quantification
