# Supplementary material for: A randomised, crossover, clinical study to assess nicotine pharmacokinetics and subjective effects of the BIDI® stick ENDS compared with combustible cigarettes and a comparator ENDS in adult smokers
Source: Harm Reduct J. 2022 Jun 2;19:57. doi: 10.1186/s12954-022-00638-0 (PMC9160848; doi:10.1186/s12954-022-00638-0)
Supplement: Supplementary file 1 — Additional file 1: Fig. S1. Image of the BIDI® Stick ENDS. Table S1. Demographic details for study subjects. aFagerström Test for Cigarette Dependence (FTCD) score at screening. bSelf-reported daily cigarette consumption at screening. Abbreviations: BMI, body mass index; min, minimum; max, maximum. Table S2. Mass loss from BIDI® Stick and JUUL ENDS during controlled and ad libitum use sessions. Data are presented in grams (g). Abbreviations: Min, minimum; max, maximum; VT, Virginia Tobacco. Table S3. Levels of Harmful and Potentially Harmful Constituents (HPHCs) in BIDI® Stick ENDS aerosol and 3R4F reference cigarette smoke. Data are per puff levels collected under International Organisation for Standardisation (ISO) conditions (55 ml puff volume, 2 second puff duration, 30 second interpuff interval) by a contract laboratory accredited under ISO 17025. Abbreviations: NA, not analysed; BDL, below detectable levels; NNK, Methyl[4-oxo-4-(pyridin-3-yl)butyl]nitrous amide; NNN, 3-[(2S)-1-Nitrosopyrrolidin-2-yl]pyridine. [file 12954_2022_638_MOESM1_ESM.docx]

Supplementary Table 1. Demographic details for study subjects. ^a^Fagerström Test for Cigarette Dependence (FTCD) score at screening. ^b^Self-reported daily cigarette consumption at screening. Abbreviations: BMI, body mass index; min, minimum; max, maximum.

| **Characteristic** | **Metric** | **Values** |
| --- | --- | --- |
| Age (years) | Mean [SD]  Median [min, max] | 39.2 [9.21]  39 [26, 55] |
| Sex (male:female) | N [%] : N [%] | 11 [61.1] : 7 [38.9] |
| Race  Caucasian | N [%] | 18 [100] |
| Weight (males; kg) | Mean [SD]  Median [min, max] | 79.3 [4.22]  78 [74, 87.5] |
| Weight (females; kg) | Mean [SD]  Median [min, max] | 66.6 [11.64]  61 [56, 90] |
| BMI (kg/m^2^) | Mean [SD]  Median [min, max] | 24.9 [3.09]  24.6 [21.1, 33.9] |
| FTCD score^a^ | Mean [SD]  Median [min, max] | 5.8 [1.66]  6 [3, 9] |
| Cigarette consumption^b^ | Mean [SD]  Median [min, max] | 16.6 [4.82]  15 [10, 30] |
| Time since smoking initiation (years) | Mean [SD]  Median [min, max] | 19.3 [7.66]  19 [9, 36] |

Supplementary Table 2. Mass loss from BIDI Stick and JUUL ENDS during controlled and *ad libitum* use sessions. Data are presented in grams (g). Abbreviations: Min, minimum; max, maximum; VT, Virginia Tobacco.

| **Use Session** | **Metric** | **BIDI^®^ Stick Arctic** | **BIDI^®^ Stick Classic** | **BIDI^®^ Stick Regal** | **BIDI^®^ Stick Solar** | **BIDI^®^ Stick Winter** | **BIDI^®^ Stick Zest** | **JUUL VT** |
| --- | --- | --- | --- | --- | --- | --- | --- | --- |
| **Controlled** | Mean | 0.0294 | 0.0448 | 0.0840 | 0.0352 | 0.0538 | 0.0637 | 0.0102 |
|  | SD | 0.0158 | 0.0561 | 0.2124 | 0.0147 | 0.1012 | 0.1119 | 0.0063 |
|  | Median | 0.0292 | 0.0337 | 0.0321 | 0.0339 | 0.0292 | 0.0327 | 0.0098 |
|  | Min, max | 0.0668, 0.0082 | 0.2536, 0.0037 | 0.9064, 0.0113 | 0.0815, 0.0136 | 0.4417, 0.0097 | 0.4937, 0.0203 | 0.0229, 0.0003 |
| ***Ad Libitum*** | Mean | 0.146 | 0.121 | 0.120 | 0.130 | 0.136 | 0.130 | 0.083 |
|  | SD | 0.0776 | 0.0545 | 0.0560 | 0.0851 | 0.0827 | 0.0888 | 0.0728 |
|  | Median | 0.145 | 0.116 | 0.131 | 0.106 | 0.153 | 0.126 | 0.079 |
|  | Min, max | 0.3309, 0.0450 | 0.1975, 0.0155 | 0.2509, 0.0248 | 0.3359, 0.0349 | 0.3160, 0.0312 | 0.4232, 0.0325 | 0.3005, 0.0070 |

**Supplementary Table 3. Levels of Harmful and Potentially Harmful Constituents (HPHCs) in BIDI^®^ Stick ENDS aerosol and 3R4F reference cigarette smoke.**  Data are per puff levels collected under International Organisation for Standardisation (ISO) conditions (55 ml puff volume, 2‑second puff duration, 30‑second interpuff interval) by a contract laboratory accredited under ISO 17025. Abbreviations: NA, not analysed; BDL, below detectable levels; NNK, Methyl[4-oxo-4-(pyridin-3-yl)butyl]nitrous amide; NNN, 3-[(2S)-1-Nitrosopyrrolidin-2-yl]pyridine.

| **HPHC** | **Units** | **Bidi**^®^ **Stick Zest** | **3R4F** |
| --- | --- | --- | --- |
| Nicotine | mg | 0.12 | 0.18 |
| Glycerol | mg | 0.63 | 0.22 |
| Propylene glycol | mg | 0.92 | NA |
| Formaldehyde | µg | 0.05 | 6.79 |
| Acetaldehyde | µg | 0.04 | 161.20 |
| Acrolein | µg | BDL | 14.40 |
| Crotonaldehyde | µg | BDL | 4.93 |
| Butyraldehyde | µg | BDL | 8.34 |
| Furfural | µg | BDL | NA |
| Benzaldehyde | µg | BDL | NA |
| Cinnamaldehyde | µg | BDL | NA |
| Vanillin | µg | BDL | NA |
| 2,3-butanedione (diacetyl) | µg | BDL | NA |
| 2,3-pentanedione (acetyl propionyl) | µg | BDL | NA |
| NNK | ng | BDL | 24.47 |
| NNN | ng | BDL | 27.4 |
| Cadmium | ng | BDL | 8.92 |
| Chromium | ng | BDL | BDL |
| Lead | ng | BDL | NA |
| Nickel | ng | BDL | BDL |
| Ethylene glycol | µg | BDL | NA |
| Diethylene glycol | µg | BDL | NA |
| Glycidol | µg | BDL | NA |
| N-butanol | µg | BDL | NA |
| Toluene | µg | BDL | 12.9 |
| Benzene | µg | BDL | 7.97 |
| Acrylonitrile | µg | BDL | 2.12 |
| Methyl acetate | µg | BDL | NA |
| Ethyl acetate | µg | BDL | NA |
| Isoamyl acetate | µg | BDL | NA |
| Isobutyl acetate | µg | BDL | NA |
| Ethyl acetoacetate | µg | BDL | NA |
| Benzyl acetate | µg | BDL | NA |
| Menthol | mg | BDL | NA |
| Propylene oxide | ng | BDL | 89.6 |

**Supplementary Figure 1. Image of the BIDI^®^ Stick ENDS.**

| 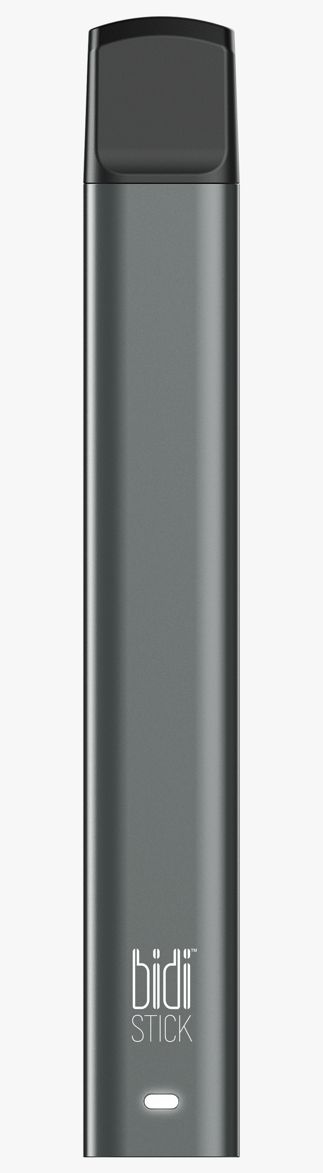 |
| --- |
